# Radiolabeled Human Monoclonal Antibody 067-213 has the Potential for Noninvasive Quantification of CD73 Expression

**DOI:** 10.3390/ijms21072304

**Published:** 2020-03-26

**Authors:** Hitomi Sudo, Atsushi B. Tsuji, Aya Sugyo, Gene Kurosawa, Yoshikazu Kurosawa, David Alexander, Hiroyuki Tsuda, Tsuneo Saga, Tatsuya Higashi

**Affiliations:** 1 Department of Molecular Imaging and Theranostics, National Institute of Radiological Sciences, National Institutes for Quantum and Radiological Science and Technology (QST-NIRS), Inage, Chiba 263-8555, Japan; sudo.hitomi@qst.go.jp (H.S.); sugyo.aya@qst.go.jp (A.S.); 2 International Center for Cell and Gene Therapy, Fujita Health University, 1-98 Dengakugakubo, Kutsukake-cho, Toyoake, Aichi 470-1192, Japan; gene@fujita-hu.ac.jp; 3 Department of Innovation Center for Advanced Medicine, Research Promotion Support Center, Fujita Health University, 1-98 Dengakugakubo, Kutsukake-cho, Toyoake, Aichi 470-1192, Japan; kurosawa@fujita-hu.ac.jp; 4 Nanotoxicology Project, Nagoya City University, 3-1 Tanabe-dohri, Mizuho-ku, Nagoya 466-8603, Japan; dalexand@phar.nagoya-cu.ac.jp (D.A.); htsuda@phar.nagoya-cu.ac.jp (H.T.); 5 Department of Advanced Medical Imaging Research, Graduate School of Medicine, Kyoto University, 54 Shogoinkawahara-cho, Sakyo-ku, Kyoto 606-8507, Japan; saga@kuhp.kyoto-u.ac.jp

**Keywords:** CD73, immune checkpoint, ectonucleotidase, extracellular adenosine, tumor microenvironment, nuclear medicine imaging, stratification of patients

## Abstract

Background: CD73 is an ectonucleotidase regulating extracellular adenosine concentration and plays an important role in adenosine-mediated immunosuppressive pathways. The efficacy of CD73-targeted therapy depends on the expression levels of CD73; therefore, monitoring CD73 status in cancer patients would provide helpful information for selection of patients who would benefit from CD73-targeted therapy. Here, we evaluated the ability of ^111^In-labeled antibody 067-213, which has high affinity for human CD73, to act as a noninvasive imaging probe. Methods: Cell binding and competitive inhibition assays for ^111^In-labeled 067-213 were conducted using MIAPaCa-2 (high CD73 expression) and A431 (low CD73 expression) cells. For in vivo assessments, biodistribution and SPECT/CT studies were conducted in MIAPaCa-2 and A431 tumor-bearing mice. To estimate the absorbed dose in humans, biodistribution and SPECT/CT studies were conducted in healthy rats. Results: ^111^In-labeled 067-213 bound to MIAPaCa-2 and A431 cells in a CD73-dependent manner and the affinity loss after ^111^In-labeling was limited. Biodistribution and SPECT/CT studies with ^111^In-labeled 067-213 in mice showed high uptake in MIAPaCa-2 tumors and lower uptake in A431 tumors. In rats, the probe did not show high uptake in normal organs, including endogenously CD73-expressing organs. The estimated absorbed doses in humans were reasonably low. Conclusions: ^111^In-labeled 067-213 showed CD73-expression-dependent tumor uptake and low uptake in normal organs and tissues. Radiolabeled 067-213 holds promise as an imaging probe for noninvasive evaluation of CD73 expression levels in patients. Our data encourage further clinical studies to clarify a role for CD73 monitoring in patients receiving CD73-targeted immune therapy.

## 1. Introduction

Extracellular adenosine is a known inhibitor of immune function: adenosine-mediated immunosuppression involves direct effects on antitumor effector cells and indirect effects on antigen-presenting cells, immunoregulatory cells such as regulatory T-cells, and myeloid-derived suppressor cells [1,2]. In contrast to normal tissues in which adenosine concentration is low, adenosine is present at high concentrations in cancer tissues [3,4] where it suppresses antitumor immune responses [1]. Hence, adenosine-mediated immunosuppression has drawn attention in the field of oncology and cancer-related immune responses [4].

CD73 is a GPI-anchored ectonucleotidase present on the cell surface membrane that dephosphorylates adenosine monophosphate to produce adenosine; CD73 activity regulates extracellular adenosine concentration [2]. The protein is expressed at high levels in many types of tumors [5,6,7], and CD73 expression levels are correlated with tumor progression and patient survival [8,9,10,11,12]. Several preclinical and clinical studies showed that CD73 is a potential biomarker for responses to chemotherapy, radiotherapy, and immune therapy [13,14,15]. In mouse models, CD73 blockade induces an immune response against tumors and suppresses tumor growth and metastasis [16]. Therefore, CD73 is currently an attractive target molecule of immune checkpoint therapy [17,18].

CD73 expression levels in tumors affect the antitumor effects induced by CD73 blockade [18,19], indicating a need to evaluate tumor CD73 expression levels for optimization of CD73-targeted immune therapy. Several types of CD73-expressing immune cells infiltrate tumors and their intratumoral distribution patterns vary [20]. Therefore, there is also a need to noninvasively determine the intratumoral distribution of tumor-infiltrating CD73-expressing cells and quantify their expression levels.

Noninvasive imaging can provide information on the expression of therapeutic targets, not only in cancer tissues but also in normal tissues, and it can predict therapeutic responses and toxicities of the targeted therapies [21]. Among various molecular imaging techniques, nuclear medicine imaging techniques such as PET and SPECT have high sensitivity and are quantifiable [22]. However, there have been no reports of CD73-targeted noninvasive imaging applicable to clinical patients. Imaging with radiolabeled anti-CD73 antibodies would provide helpful information for the stratification of patients for CD73-targeted therapy.

Using our highly efficient antibody screening method, we previously isolated a human monoclonal antibody, 067-213, from a large-scale human antibody library we constructed, AIMS5, that recognizes human CD73 with high affinity [23,24]. In the present study, the antibody 067-213 was labeled with the gamma-emitter In-111 and the in vitro properties of ^111^In-labeled 067-213 were evaluated by cell binding and competitive inhibition assays. Next, the in vivo ability of ^111^In-067-213 to be used as a noninvasive imaging probe was assessed by biodistribution and SPECT/CT imaging studies in two different tumor-bearing mice. Lastly, to estimate the risk of radiation-induced toxicity in humans, a dosimetry study was conducted based on the biodistribution of ^111^In-067-213 in normal rats: the antibody 067-213 cross-reacts with CD73 expressed in rats.

## 2. Results

### 2.1. Binding Properties of Anti-CD73 Antibody 067-213

**Figure 1:**
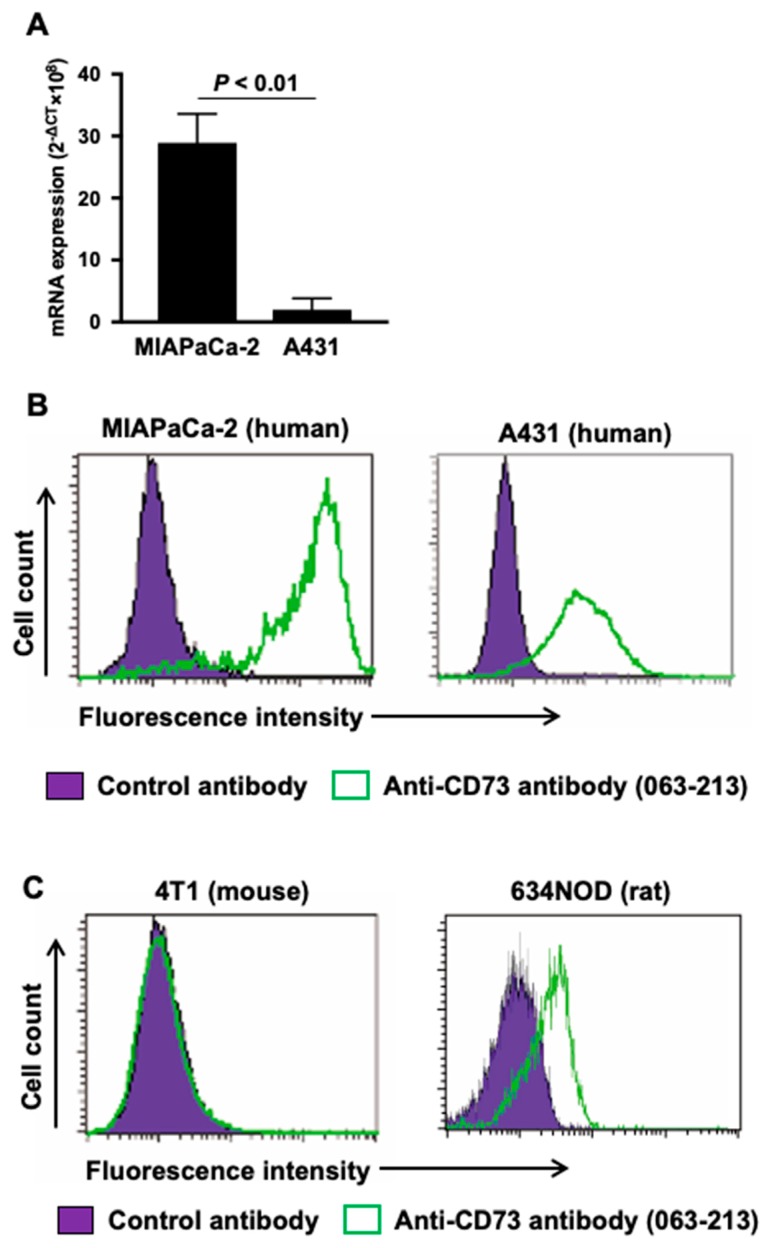
(**A**) CD73 mRNA expression analysis in MIAPaCa-2 and A431. (**B**) Flow cytometric analysis of anti-CD73 antibody 067-213 to two human cancer cell lines MIAPaCa-2 and A431. (**C**) Flow cytometric analysis of anti-CD73 antibody 067-213 to mouse and rat cell lines 4T1 and 634NOD. Green, anti-CD73 antibody; purple, control antibody.

To select appropriate human cancer cell lines for models of high and low CD73 expression, quantitative real-time RT-PCR and flow cytometry analyses were conducted. The two human cancer cell lines MIAPaCa-2 [25] and A431 [26] are reported to be CD73 positive. Quantitative real-time RT-PCR analysis confirmed that CD73 mRNA expression in MIAPaCa-2 cells was 20-hold higher than that in A431: *p* < 0.01 (Figure 1A). Flow cytometric analysis with the antibody 067-213 demonstrated higher fluorescence intensity in MIAPaCa-2 cells, mean fluorescence intensity = 105.5, compared with A431, mean fluorescence intensity = 23.6 (Figure 1B). These results are consistent with antibody 067-213 binding to these human cancer cells being dependent on CD73 expression levels, and affirm the use of MIAPaCa-2 as a model of CD73 high expression and A431 as a model of CD73 low expression in experiments to evaluate the in vitro and in vivo properties of radiolabeled 067-213 as an imaging probe.

To select an appropriate animal to predict the biodistribution of radiolabeled 067-213 in humans, flow cytometric analyses were conducted using the murine cancer cell line 4T1 and the rat cancer cell line 634NOD. Although 4T1 cells were reported to be CD73 positive as determined by flow cytometry with another anti-CD73 antibody [27], no peak shift with 067-213 was observed (Figure 1C). In contrast, the antibody 067-213 induced a peak shift in 634NOD cells (mean fluorescence intensity = 20.0) (Figure 1C). These results are consistent with antibody 067-213 reacting with CD73 expressed in rat cancer cells but not in murine cancer cells. Hence, rats were selected to evaluate the biodistribution of radiolabeled 067-213 in normal organs and tissues for estimation of risk of human toxicity.

### 2.2. In vitro Characteristics of ^111^In-labeled Anti-CD73 Antibody

**Figure 2:**
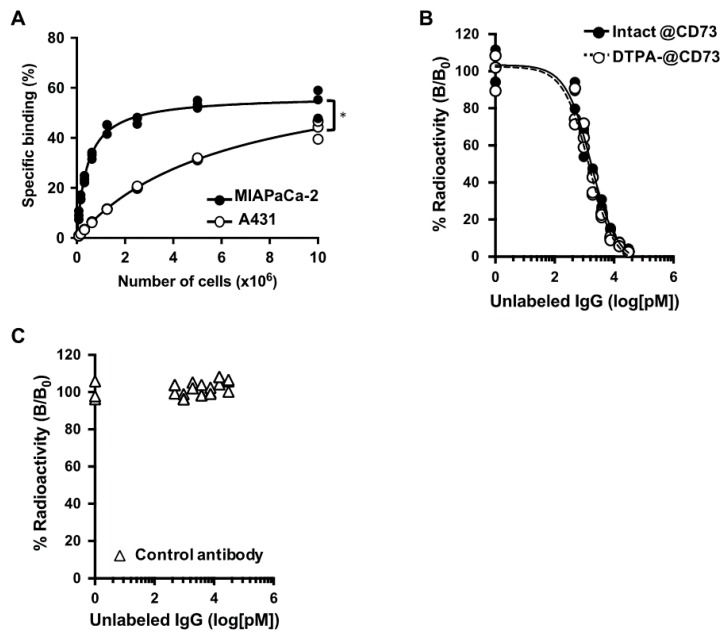
(**A**) Cell binding assay of ^111^In-labeled anti-CD73 antibody 067-213 with MIAPaCa-2 (CD73 high, black circles) and A431 (CD73 low, white circles) cells. * *p* < 0.05. (**B**) Competitive inhibition assay of ^111^In-labeled anti-CD73 antibody to MIAPaCa-2 cells with intact anti-CD73 antibody (black circles) and CHX-A″-DTPA-conjugated CD73 antibody (white circles) as a competitor. (**C**) Competitive inhibition assay of ^111^In-labeled anti-CD73 antibody to MIAPaCa-2 cells with control antibody as the competitor.

Figure 2A shows the results of cell binding assays with MIAPaCa-2 (CD73 high) and A431 (CD73 low) cells. Binding of ^111^In-labeled 067-213 to both cell types increased in a cell concentration-dependent manner. The binding to MIAPaCa-2 was significantly higher than that to A431 (*p* < 0.05), especially at lower cell concentrations. The maximum specific binding to MIAPaCa-2 and A431 cells was 54.0 ± 5.7% and 43.5 ± 3.6%, respectively, at 1 × 10^7^ cells.

The results of competitive inhibition assays using MIAPaCa-2 cells are shown in Figure 2B. The binding affinities (K_d_) of intact 067-213 and *p*-SCN-Bn-CHX-A″-DTPA (CHX-A″-DTPA)-conjugated 067-213 were estimated to be 0.85 and 0.72 nM, respectively.

Control antibody (human IgG) did not inhibit the binding of the ^111^In-labeled 067-213 to MIAPaCa-2 cells (Figure 2C). These findings indicate that impairment of the affinity of 067-213 for CD73 due to the CHX-A″-DTPA conjugation procedure was minimal, and that the chelate-conjugated 067-213 would be applicable for in vivo experiments.

### 2.3. Biodistribution of ^111^In-Labeled Anti-CD73 Antibody in Tumor-Bearing Mice

Table 1 shows the biodistribution of ^111^In-labeled 067-213 in MIAPaCa-2- and A431-tumor-bearing mice. The ^111^In-labeled 067-213 uptake in MIAPaCa-2 tumors was significantly higher than that in A431 tumors at every time point (*p* < 0.05 at 6 h; *p* < 0.01 at 24, 48, and 96 h). The MIAPaCa-2 tumor uptake markedly increased with time and reached the highest uptake level of 60.7 ± 7.2 percent of the injected radioactivity dose per gram (% ID/g) at 96 h. A431 tumor uptake also showed a slight increase over time but the highest value was only 8.8 ± 0.7% ID/g at 96 h.

In A431-tumor-bearing mice, ^111^In-labeled antibody uptake in healthy organs tended to be higher than in MIAPaCa-2-tumor-bearing mice. The uptake in the spleen, kidney, and bone in A431-tumor-bearing mice was significantly higher than in MIAPaCa-2-tumor-bearing mice at 6 and 24 h (*p* < 0.05 for spleen and kidney at 6 h; *p* < 0.01 for bone at 6 h; and *p* < 0.01 for spleen, kidney, and bone at 24 h). Similarly, uptake in blood, brain, lung, pancreas, and intestine in A431-tumor-bearing mice was significantly higher than in MIAPaCa-2-tumor-bearing mice at 48 h (*p* < 0.05 for brain and lung; *p* < 0.01 for blood, pancreas, and intestine). At 96 h after injection, only blood and kidney in the A431-tumor-bearing mice had a significantly higher probe uptake than the MIAPaCa-2-tumor-bearing mice (*p* < 0.01). Although, at this time point, probe uptake in the liver of the MIAPaCa-2-tumor-bearing mice was higher than in the A431-tumor-bearing mice (*p* < 0.05).

### 2.4. SPECT/CT Imaging of ^111^In-Labeled Anti-CD73 Antibody in Tumor-Bearing Mice

**Figure 3:**
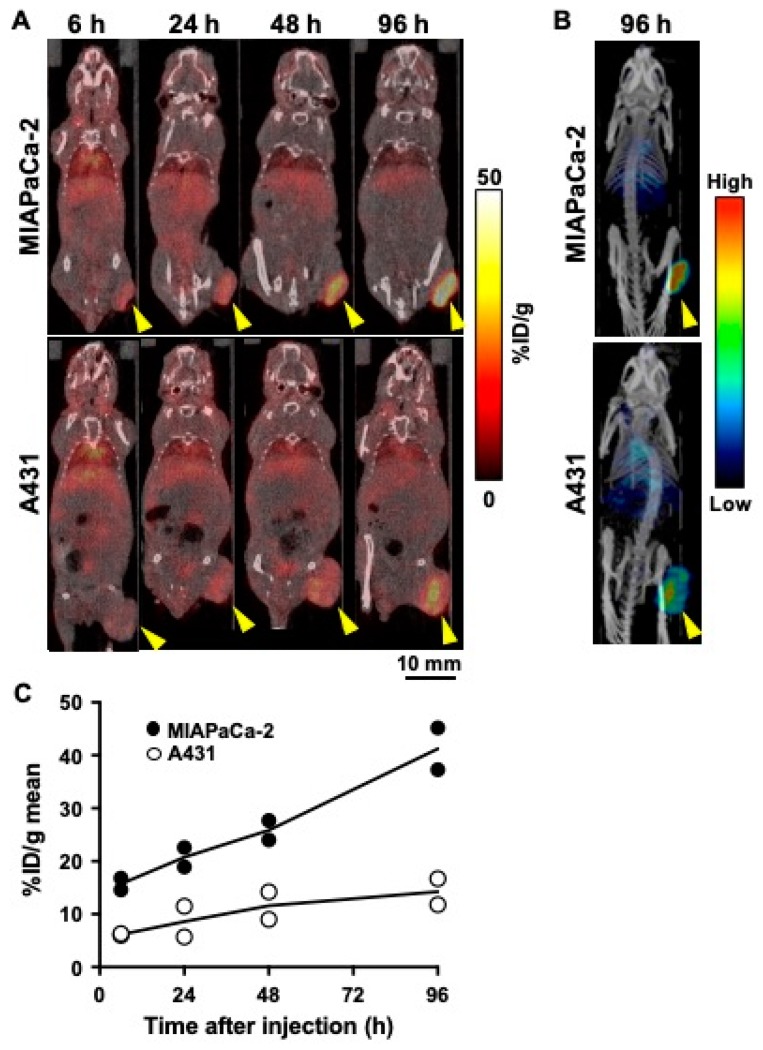
Temporal SPECT/CT imaging of ^111^In-labeled anti-CD73 antibody 067-213 in tumor-bearing mice. (**A**) Representative SPECT/CT images at 6, 24, 48, and 96 h after intravenous injection (1.85 MBq). Arrowheads indicate tumors. (**B**) Maximum intensity projection images at 96 h in the same mice as in **A**. (**C**) Temporal change of radioactivity of ^111^In-labeled anti-CD73 antibody in the MIAPaCa-2 (black circles) and A431 (white circles) tumors. The uptake data were acquired from SPECT/CT images (*n* = 2).

SPECT/CT imaging of ^111^In-labeled 067-213 visualized MIAPaCa-2 tumors more clearly than A431 tumors (Figure 3A,B). Quantification of SPECT data indicated that MIAPaCa-2 tumor uptake increased with time and reached the highest value of 41.3 ± 5.6% ID/g at 96 h after injection (Figure 3C). In contrast, A431 tumor uptake showed only a slight increase with time and the highest value was 14.3 ± 3.5% ID/g at 96 h (Figure 3C). These observations are consistent with the results of the biodistribution studies in tumor-bearing mice (Table 1).

### 2.5. Biodistribution of ^111^In-Labeled Anti-CD73 Antibody in Healthy Rats

Table 2 shows the biodistribution of ^111^In-labeled 067-213 in healthy rats. The radioactivity of ^111^In-labeled 067-213 was mainly present in the blood at 6 h after injection (11.9 ± 1.7% ID/g). Labeled probe in the blood decreased over time and reached 4.2 ± 0.6% ID/g at 96 h. Uptake of ^111^In-labeled 067-213 was low to moderate in all organs and, similarly to the blood, the presence of the probe in healthy organs decreased with time. This is consistent with the findings that compared to a variety of tumors constitutive expression of CD73 in healthy tissues is relatively low [28], and that specific uptake of ^111^In-labeled 067-213 in healthy tissues is minor. The biodistribution data in rats was converted into values for an adult human male, and the absorbed doses were estimated. The absorbed dose for the total body was 0.13 millisievert per megabecquerel of the probe (mSv/MBq) and the red marrow absorbed dose was 0.12 mSv/MBq (Table 3).

### 2.6. SPECT/CT Imaging of ^111^In-labeled anti-CD73 antibody in a healthy rat

**Figure 4:**
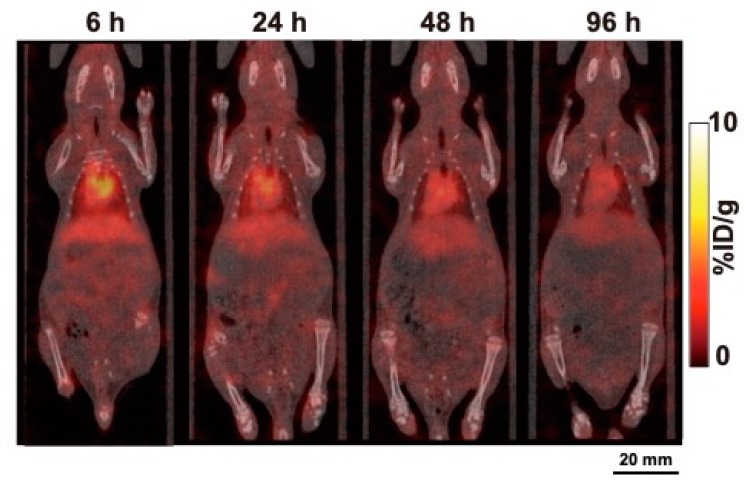
Temporal SPECT/CT imaging of ^111^In-labeled anti-CD73 antibody 067-213 in a healthy rat. Representative SPECT/CT images at 6, 24, 48, and 96 h after intravenous injection (2.59 MBq).

Representative SPECT/CT images of ^111^In-labeled 067-213 at 6, 24, 48, and 96 h after injection are shown in Figure 4. Imaging showed marked accumulation of ^111^In-labeled 067-213 in the heart and liver at 6 h, with the presence of the probe in these organs decreasing with time. No marked accumulation of the probe in the other organs was observed in the SPECT/CT images.

## 3. Discussion

Noninvasive imaging of immunotherapy targets enables prediction of responses and toxicities of the designated immunotherapy [21]. In the present study, we radiolabeled a human anti-CD73 monoclonal antibody (antibody 067-213) [24] with the gamma-emitter In-111, and evaluated the in vitro and in vivo properties of ^111^In-labeled 067-213 as an imaging probe in tumor-bearing mice and healthy rats. Our findings are consistent with the proposition that radiolabeled 067-213 has the potential to noninvasively quantify CD73 expression levels in cancer patients with reasonably low radiation doses. To the best of our knowledge, ^111^In-labeled 067-213 is the first imaging probe applicable to clinical patients.

Cell binding assays showed that ^111^In-labeled 067-213 had higher binding to MIAPaCa-2 cells than to A431 cells. Since MIAPaCa-2 cells express high levels of CD73 and A431 cells express low levels of CD73, these results indicate that the binding of ^111^In-labeled 067-213 reflected CD73 expression levels. Competitive inhibition assays showed that CD73 affinity loss after the labeling procedure was limited, signifying that chelate-conjugated 067-213 retained its high binding affinity to CD73. These data indicate that radiolabeled 067-213 is potentially suitable as an in vivo imaging probe.

Biodistribution studies with ^111^In-labeled 067-213 in two different tumor mouse models showed much higher uptake of ^111^In-labeled 067-213 in MIAPaCa-2 tumors compared with A431 tumors. Serial SPECT/CT imaging with ^111^In-labeled 067-213 confirmed the biodistribution analysis: ^111^In-labeled 067-213 uptake was higher in tumors expressing higher levels of CD73 and lower in tumors expressing lower levels of CD73.

In order to bring this promising probe to the clinic, extrapolation of preclinical data to humans is necessary. Therefore, a suitable model should be selected for the evaluation of whole-body distribution of the probe. Although the biodistribution and SPECT/CT studies with ^111^In-labeled 067-213 showed low uptake in normal organs in mice, our flow cytometric analysis in murine 4T1 cells showed that 067-213 does not recognize murine CD73. Therefore, the biodistribution of ^111^In-labeled 067-213 in mice may not reflect the biodistribution in humans. In contrast, flow cytometric analysis of rat 634NOD cells indicated that 067-213 reacts with rat CD73, suggesting that rats are a suitable animal model to use to estimate the distribution pattern of ^111^In-labeled 067-213 in normal human tissues and for risk assessment in humans after administration of ^111^In-labeled 067-213. Biodistribution and SPECT/CT imaging analyses showed low to moderate uptake of ^111^In-labeled 067-213 in normal organs and tissues in healthy rats, including CD73-positive organs such as the lungs, liver, spleen, intestines, kidneys, and bone marrow [28]. These findings suggest that the expression levels of CD73 in normal organs and tissues will have a minimal effect on immuno-SPECT/CT imaging in humans.

Based on the biodistribution data in rats, the total body absorbed dose in humans was estimated to be 0.13 mSv/MBq and the red marrow absorbed dose was estimated to be 0.12 mSv/MBq. Estimates of the absorbed dose in human organs, including CD73-expressing organs, project low to moderate absorbed doses. The estimated absorbed doses of ^111^In-labeled 067-213 in humans are similar to those of ^111^In-labeled Zevalin, which is used as a tracer to assess biodistribution and dosimetry of the therapeutic agent ^90^Y-labeled Zevalin [29,30]. These data suggest that ^111^In-labeled 067-213 is a feasible imaging probe for the detection and quantitative analysis of CD73 expression.

The present study employed the MIAPaCa-2 tumor model, which is derived from pancreatic cancer. Pancreatic cancer has a rich stroma that plays a key role in pancreas cancer progression and treatment resistance [31,32]. Our imaging probe might be useful for investigating the role of CD73 in these mechanisms. Using pancreatic cancer cells, including MIAPaCa-2 cells, Ryzhov et al. showed CD73-positive myeloid cells are immunosuppressive, proangiogenic, and tumor-promoting [33]. Messaoudi et al. demonstrated that CD73 expression on cancer cells was higher in tumors having lymph node metastasis compared with tumors having no lymph node metastasis [34]. Solimando and colleagues found increased WNT activation in lymphatic dissemination and corroborated immune-microenvironment interaction in nodal positive disease using MIAPaCa-2 and PANC-1 cells [35]. Taken together, these studies suggest that CD73-mediated adenosine signaling, which is critical for cell adhesion and promotes localization of E-cadherin and β-catenin to the cell membrane, might modulate β-catenin/WNT signaling, which in turn can suppress chemokine production from tumor cells and prevent T-cell infiltration. Noninvasive imaging of CD73 in MIAPaCa-2 tumor models might provide new insights into the role of CD73 in the immune microenvironment in pancreatic cancer.

There are limitations in this initial study. First, while subcutaneously implanted BALB/c-nu/nu mice are well-established models, the tumor microenvironment characteristics differ from those in humans. This needs to be considered when interpreting our findings. Second, the present study employed only two tumors having different CD73 expression levels. Further preclinical studies are required to affirm that ^111^In-labeled 067-213 has the potential to evaluate CD73 expression levels in tumors. Third, the biodistribution of ^111^In-labeled 067-213 in rats was used to estimate the absorbed doses of radiation in humans. It is likely that there are differences in biodistribution of ^111^In-labeled 067-213 in humans and rodents. Therefore, dosimetry studies in a few selected patients will be required for assessment of human exposure and risk before expanded clinical studies can be considered.

In conclusion, the present biodistribution and SPECT/CT studies in tumor-bearing mice suggest that the radiolabeled anti-CD73 human monoclonal antibody 067-213 has the potential to noninvasively determine CD73 expression levels in cancer patients. Based on its biodistribution in rats, the estimated absorbed doses in humans suggest that SPECT/CT imaging with ^111^In-labeled 067-213 in cancer patients is reasonable. These findings indicate that further pre-clinical studies to ascertain the suitability of ^111^In-labeled 067-213 as a probe to noninvasively monitor CD73 status in cancer patients receiving CD73-targeted immune checkpoint therapy are appropriate.

## 4. Materials and Methods

### 4.1. Cell Culture

The human epidermoid carcinoma cell line A431, the human pancreatic cancer cell line MIAPaCa-2, and the mouse breast carcinoma cell line 4T1 were obtained from ATCC (Manassas, VA, USA). The 634NOD cell line was originally established from a pancreatic carcinoma in an HRAS^G12V^ transgenic rat [36]. A431 and MIAPaCa-2 cells were cultured in Dulbecco modified Eagle medium (Wako Pure Chemical Industries, Tokyo, Japan) containing 10% fetal bovine serum (FBS) (Life Technologies, Carlsbad, CA, USA) in 5% CO_2_ at 37 °C. 4T1 cells were cultured in RPMI-1640 (Wako Pure Chemical Industries) containing 10% FBS in 5% CO_2_ at 37 °C. 634NOD cells were cultured in RPMI-1640 containing 10% FBS, BEGM SingleQuots endothelial cell growth medium (Lonza, Basel, Switzerland), and antibiotic antimycotic solution (GE Healthcare, Little Chalfont, UK) in 5% CO_2_ at 37 °C.

### 4.2. Antibodies

The human monoclonal antibody 067-213 recognizing human CD73 was isolated from the phage library of human monoclonal antibodies AIMS-5 [37] using the ICOS method [23] as previously described [24,26]. A human IgG as a control antibody was purchased from Sigma-Aldrich (St. Louis, MO, USA).

### 4.3. Quantitative Real-Time RT-PCR

First-strand cDNAs were synthesized from human cell lines using a FastLane Cell cDNA kit (Qiagen, Hilden, Germany). Predesigned and preoptimized TaqMan probes to detect CD73 and 18S rRNA were purchased from Thermo Fisher Scientific (Waltham, MA, USA). Real-time RT-PCR was conducted in triplicate using a Premix Ex Taq reagent (Takara Bio, Otsu, Japan) and Mx3000P qPCR systems (Agilent Technologies, Santa Clara, CA, USA). Gene expression levels were normalized to 18S rRNA expression in each sample. Three independent experiments were conducted.

### 4.4. Flow Cytometry

Cells (2 × 10^6^) were incubated with anti-CD73 antibody 067-213 (10 µg/mL) or human IgG (10 µg/mL) for 60 min at room temperature. After being washed with ice-cold PBS (Wako Pure Chemical Industries), the cells were incubated with Alexa Fluor 488-labeled anti-human IgG (10 µg/mL) (Invitrogen, Carlsbad, CA, USA) for 30 min at room temperature. The stained cells were analyzed using a FACS Calibur flow cytometer and CellQuest software (Becton Dickinson Biosciences, Franklin Lakes, NJ, USA).

### 4.5. Radiolabeling

The anti-CD73 antibody 067-213 was conjugated with *p*-SCN-Bn-CHX-A″-DTPA (CHX-A″-DTPA; Macrocyclics, Dallas, TX, USA) as described previously [38]. Briefly, the antibody (5 mg/mL) was reacted with an equal number of moles of CHX-A″-DTPA in 50 mM borate buffer (pH 8.5) for 16 h at 37 °C. The conjugation ratio of CHX-A″-DTPA to antibody was estimated to be approximately 1.7 as determined by cellulose acetate electrophoresis. The CHX-A″-DTPA-conjugated antibody was purified by elution with 0.1 M acetate buffer (pH 6.0) using a Sephadex G-50 (GE Healthcare) column. ^111^InCl_3_ (Nihon Medi-Physics, Tokyo, Japan) was mixed with the CHX-A″-DTPA-antibody conjugate in 1.0 M acetate buffer (pH 6.0) and incubated for 30 min at room temperature. The radiolabeled antibody was eluted with 0.1 M acetate buffer (pH 6.0) using a Sephadex G-50 column for purification. The specific activity of the ^111^In-labeled anti-CD73 antibody was approximately 45 kBq/µg. The labeling yields were approximately 60%, and the radiochemical purity was more than 97%.

### 4.6. Cell Binding and Competitive Inhibition Assays

For cell binding assays, A431 and MIAPaCa-2 cells (5.0 × 10^6^, 2.6 × 10^6^, 1.3 × 10^6^, 6.3 × 10^5^, 3.1 × 10^5^, 1.6 × 10^5^, 7.8 × 10^4^, and 3.9 × 10^4^) in PBS with 1% BSA (Sigma-Aldrich) were incubated with ^111^In-labeled anti-CD73 antibody 067-213 on ice for 60 min. After washing, cell-bound radioactivity was measured using a γ-counter (ARC-370M, Aloka, Tokyo, Japan). For competitive inhibition assays, MIAPaCa-2 cells (2.0 × 10^6^) in PBS with 1% BSA were incubated with ^111^In-labeled anti-CD73 antibody in the presence of varying concentrations of intact anti-CD73 antibody, CHX-A″-DTPA-conjugated anti-CD73 antibody, or control antibody (0, 0.5, 0.9, 1.9, 3.8, 7.6, 15.2, and 30.3 nmol/L) on ice for 60 min. After washing, cell-bound radioactivity was measured with a γ-counter. The dissociation constant was estimated by applying data to a one-site competitive binding model using GraphPad Prism software (ver. 7.0; GraphPad Software, La Jolla, CA, USA).

### 4.7. Animals

The animal experimental protocol was approved by the Animal Care and Use Committee of the National Institute of Radiological Sciences, and all animal experiments were conducted following the National Institute of Radiological Sciences Institutional Guidelines regarding Animal Care and Handling. Female nude mice (BALB/c-*nu/nu*, 6 weeks old) and rats (Jcl: SD, 4 weeks old) were obtained from CLEA Japan (Tokyo, Japan). To establish tumor mouse models, A431 (3 × 10^6^) or MIAPaCa-2 (5 × 10^6^) cells were subcutaneously inoculated into mice under isoflurane anesthesia. Pelleted food (Funabashi Farm, Chiba, Japan) and water were provided ad libitum. The animal room had a controlled temperature (23 °C ± 3 °C), humidity (50% ± 20%), and light–dark cycle (12 h on and 12 h off).

### 4.8. Biodistribution of ^111^In-Labeled Antibody and Absorbed Dose Estimation

Nude mice bearing A431 or MIAPaCa-2 tumors (*n* = 5 per each time-point) and rats (n = 4 per each time-point) were intravenously injected with ^111^In-labeled anti-CD73 antibody 067-213 (3.7 kBq/g body weight). The total injected protein dose was adjusted to 1.0 µg per g body weight by adding unlabeled antibody. The mice and rats were sacrificed at 6, 24, 48, and 96 h after injection. Tissues and organs of interest were dissected, weighed, and radioactivity was measured with a γ-counter. Radiotracer uptake is represented as the percentage injected radioactivity dose per gram (% ID/g). Absorbed doses were estimated with OLINDA/EXM version 1.0 software (Vanderbilt University, Nashville, TN, USA) as previously described [39]. The biodistribution data in rats was converted to that in a reference human male [40].

### 4.9. SPECT/CT Imaging

Two nude mice bearing A431 subcutaneous tumors and two nude mice bearing MIAPaCa-2 subcutaneous tumors were injected intravenously with 1.85 MBq ^111^In-labeled anti-CD73 antibody 067-213. The total injected protein dose was adjusted to 20 µg per mouse by adding unlabeled antibody. A single rat was injected intravenously with 37 kBq/g body weight ^111^In-labeled anti-CD73 antibody. The total injected protein dose was adjusted to 2 µg per g body weight by adding unlabeled antibody. At 6, 24, 48, and 96 h after injection, the mice and the rat were anesthetized by isoflurane inhalation, and SPECT/CT data were acquired using a VECTor/CT SPECT/CT Pre-Clinical Imaging system with a multi-pinhole collimator (MILabs, Utrecht, the Netherlands). Following SPECT imaging, CT data were acquired with an X-ray source set at 60 kVp and 615 µA. SPECT images were reconstructed using a pixel-based ordered-subsets expectation-maximization algorithm with 2 subsets and 8 iterations on a 0.8-mm voxel grid without attenuation correction. The volume of interest was manually drawn over tumors, and tracer uptake was quantified by PMOD data analysis software (version 3.4, PMOD Technology, Zürich, Switzerland). CT images were reconstructed using a filtered back-projection algorithm for cone beam. Merged images of SPECT and CT were obtained using PMOD software.

### 4.10. Statistical Analysis

Data are expressed as the means ± standard deviation. Gene expression, cell binding, and radiotracer uptake data followed a Gaussian distribution: the data shown in Figure 1 and Table 1 were evaluated by unpaired *t*-test and the data shown in Figure 2 was evaluated by two-way ANOVA using GraphPad Prism 7 software (GraphPad Software, San Diego, CA, USA). *p* < 0.05 was considered statistically significant in all experiments.

## Figures and Tables

**Table 1:** Biodistribution of ^111^In-labeled anti-CD73 antibody 063-213 in tumor-bearing mice. Data are represented as the mean of %ID/g ± standard deviation (*n* = 5). * *p* < 0.05, ** *p* < 0.01 vs. A431-bearing mice at each time point.

	6 h	24 h	48 h	96 h
MIAPaCa-2				
Blood	25.4 ± 3.6	21.0 ± 2.6	16.0 ± 2.4 **	14.0 ± 1.8 **
Brain	0.7 ± 0.3	0.5 ± 0.1	0.4 ± 0.1 *	0.4 ± 0.1
Heart	6.2 ± 0.7	5.4 ± 0.9	4.2 ± 0.9	4.0 ± 0.5
Lung	9.5 ± 1.2	8.2 ± 1.3	7.2 ± 0.9 *	7.4 ± 0.8
Liver	8.8 ± 0.6	8.1 ±1.6	6.8 ± 0.5	6.6 ± 1.3 *
Spleen	4.5 ± 0.6 *	4.5 ± 0.8 **	4.6 ± 0.7	4.4 ± 0.6
Pancreas	1.9 ± 0.2	2.0 ± 0.3	1.6 ± 0.2 **	1.6 ± 0.2
Stomach	2.5 ± 0.4	2.2 ± 0.5	1.9 ± 0.3	2.0 ± 0.3
Intestine	3.2 ± 0.5	2.7 ± 0.3	2.0 ± 0.2 **	2.0 ± 0.4
Kidney	7.1 ± 1.4 *	5.2 ± 0.8 **	5.1 ± 0.6	4.6 ± 0.5 **
Muscle	1.1 ± 0.2	1.5 ± 0.3	1.3 ± 0.2	1.1 ± 0.2
Bone	2.2 ± 0.4 **	1.9 ± 0.3 **	1.7 ± 0.3 **	1.9 ± 0.3
Tumor	11.0 ± 5.6 *	21.2 ± 2.5 **	53.9 ± 21.0 **	60.7 ± 7.2 **
A431				
Blood	28.5 ± 1.6	24.6 ± 3.0	21.5 ± 1.8	18.6 ± 2.4
Brain	1.0 ± 0.2	0.6 ± 0.1	0.6 ± 0.1	0.5 ± 0.1
Heart	6.8 ± 0.5	6.3 ± 0.7	5.2 ± 0.8	4.1 ± 0.2
Lung	10.0 ± 0.9	9.6 ± 1.5	8.9 ± 1.0	7.0 ± 0.5
Liver	9.8 ± 1.1	7.5 ±1.5	6.5 ± 0.8	4.9 ± 0.7
Spleen	7.3 ± 2.0	6.5 ± 0.5	6.6 ± 1.8	4.8 ± 0.2
Pancreas	2.1 ± 0.3	2.4 ± 0.2	2.1 ± 0.1	1.7 ± 0.2
Stomach	2.8 ± 0.4	2.6 ± 0.5	2.3 ± 0.4	2.0 ± 0.2
Intestine	3.7 ± 0.1	2.9 ± 0.5	2.9 ± 0.4	2.2 ± 0.3
Kidney	9.0 ± 0.6	7.2 ± 0.6	6.3 ± 1.0	5.7 ± 0.3
Muscle	1.0 ± 0.2	1.4 ± 0.1	1.4 ± 0.2	1.2 ± 0.1
Bone	3.1 ± 0.3	2.9 ± 0.3	2.9 ± 0.5	2.1 ± 0.2
Tumor	3.5 ± 0.4	6.9 ± 0.5	8.1 ± 1.1	8.8 ± 0.7

**Table 2:** Biodistribution of ^111^In-labeled anti-CD73 antibody 063-213 in healthy rats.

	6 h	24 h	48 h	96 h
Blood	11.9 ± 1.7	7.6 ± 1.7	5.9 ± 0.6	4.2 ± 0.6
Thymus	1.0 ± 0.2	1.0 ± 0.1	0. 8 ± 0.1	0.7 ± 0.1
Lung	4.2 ± 0.5	3.0 ± 0.5	2.5 ± 0.2	1.9 ± 0.1
Liver	3.7 ± 0.5	2.6 ± 0.5	1.9 ± 0.3	1.6 ± 0.1
Spleen	2.6 ± 0.2	2.4 ± 0.2	2.0 ± 0.3	1.5 ± 0.2
Pancreas	1.1 ± 0.1	1.2 ± 0.1	0.8 ± 0.1	0.8 ± 0.05
Stomach	1.1 ± 0.1	0.9 ± 0.1	0.7 ± 0.1	0.6 ± 0.03
Intestine	2.0 ± 0.2	1.4 ± 0.2	1.2 ± 0.1	0.9 ± 0.04
Kidney	3.8 ± 0.4	3.0 ± 0.4	2.6 ± 0.3	2.6 ± 0.2
Muscle	0.6 ± 0.1	0.9 ± 0.1	0.8 ± 0.1	0.6 ± 0.04
Bone	1.8 ± 0.3	1.4 ± 0.3	1.1 ± 0.1	1.0 ± 0.1

Data are represented as the mean of %ID/g ± standard deviation (*n* = 4).

**Table 3:** Estimated absorbed doses of ^111^In-labeled anti-CD73 antibody 063-213 in major organs and tissues in a human adult male based on biodistribution in rats.

Target organ	Absorbed Dose
	(mSv/MBq)
Adrenals	0.16
Brain	0.12
Breasts	0.10
Gallbladder wall	0.17
Lower large intestine wall	0.16
Small intestine	0.19
Stomach wall	0.14
Upper large intestine wall	0.17
Heart wall	0.16
Kidneys	0.15
Liver	0.17
Lungs	0.18
Muscle	0.11
Pancreas	0.15
Red marrow	0.12
Osteogenic cells	0.20
Skin	0.08
Spleen	0.11
Testes	0.11
Thymus	0.15
Thyroid	0.13
Urinary bladder wall	0.15
Uterus	0.17
Total body	0.13
Effective dose equivalent	0.15
Effective dose	0.14

## Abbreviations

ANOVA	analysis of variance
CT	computed tomography
ID/g	injected radioactivity dose per gram
mRNA	messenger ribonucleic acid
mSV/MBq	millisievert per megabequrel
*p*-SCN-Bn-CHX-A″-DTPA	[(*R*)-2-Amino-3-(4-isothiocyanatophenyl)propyl]-trans-(*S*,*S*)-cyclohexane-1,2-diamine-pentaacetic acid
PBS	phosphate-buffered saline
PET	positron emission tomography
rRNA	ribosomal ribonucleic acid
RT-PCR	reverse transcription polymerase chain reaction
SPECT	single photon emission computed tomography

## References

[1] Ohta A. (2016). A Metabolic Immune Checkpoint: Adenosine in Tumor Microenvironment. Front. Immunol..

[2] Whiteside T.L. (2017). Targeting adenosine in cancer immunotherapy: A review of recent progress. Expert. Rev. Anticancer Ther..

[3] Blay J., White T.D., Hoskin D.W. (1997). The extracellular fluid of solid carcinomas contains immunosuppressive concentrations of adenosine. Cancer Res..

[4] Antonioli L., Blandizzi C., Pacher P., Hasko G. (2013). Immunity, inflammation and cancer: A leading role for adenosine. Nat. Rev. Cancer.

[5] Hausler S.F., Montalban del Barrio I., Strohschein J., Chandran P.A., Engel J.B., Honig A., Ossadnik M., Horn E., Fischer B., Krockenberger M. (2011). Ectonucleotidases CD39 and CD73 on OvCA cells are potent adenosine-generating enzymes responsible for adenosine receptor 2A-dependent suppression of T cell function and NK cell cytotoxicity. Cancer Immunol. Immunother..

[6] Loi S., Pommey S., Haibe-Kains B., Beavis P.A., Darcy P.K., Smyth M.J., Stagg J. (2013). CD73 promotes anthracycline resistance and poor prognosis in triple negative breast cancer. Proc. Natl. Acad. Sci. USA.

[7] Bastid J., Regairaz A., Bonnefoy N., Dejou C., Giustiniani J., Laheurte C., Cochaud S., Laprevotte E., Funck-Brentano E., Hemon P. (2015). Inhibition of CD39 enzymatic function at the surface of tumor cells alleviates their immunosuppressive activity. Cancer Immunol. Res..

[8] Liu N., Fang X.D., Vadis Q. (2012). CD73 as a novel prognostic biomarker for human colorectal cancer. J. Surg. Oncol..

[9] Wu X.R., He X.S., Chen Y.F., Yuan R.X., Zeng Y., Lian L., Zou Y.F., Lan N., Wu X.J., Lan P. (2012). High expression of CD73 as a poor prognostic biomarker in human colorectal cancer. J. Surg. Oncol..

[10] Lu X.X., Chen Y.T., Feng B., Mao X.B., Yu B., Chu X.Y. (2013). Expression and clinical significance of CD73 and hypoxia-inducible factor-1alpha in gastric carcinoma. World J. Gastroenterol..

[11] Xiong L., Wen Y., Miao X., Yang Z. (2014). NT5E and FcGBP as key regulators of TGF-1-induced epithelial-mesenchymal transition (EMT) are associated with tumor progression and survival of patients with gallbladder cancer. Cell Tissue Res..

[12] Supernat A., Markiewicz A., Welnicka-Jaskiewicz M., Seroczynska B., Skokowski J., Sejda A., Szade J., Czapiewski P., Biernat W., Zaczek A. (2012). CD73 expression as a potential marker of good prognosis in breast carcinoma. Appl. Immunohistochem. Mol. Morphol..

[13] Cushman S.M., Jiang C., Hatch A.J., Shterev I., Sibley A.B., Niedzwiecki D., Venook A.P., Owzar K., Hurwitz H.I., Nixon A.B. (2015). Gene expression markers of efficacy and resistance to cetuximab treatment in metastatic colorectal cancer: Results from CALGB 80203 (Alliance). Clin. Cancer Res..

[14] Morello S., Capone M., Sorrentino C., Giannarelli D., Madonna G., Mallardo D., Grimaldi A.M., Pinto A., Ascierto P.A. (2017). Soluble CD73 as biomarker in patients with metastatic melanoma patients treated with nivolumab. J. Transl. Med..

[15] Nguyen A.M., Zhou J., Sicairos B., Sonney S., Du Y. (2020). Upregulation of CD73 Confers Acquired Radioresistance and is Required for Maintaining Irradiation-selected Pancreatic Cancer Cells in a Mesenchymal State. Mol. Cell. Proteomics.

[16] Stagg J., Divisekera U., McLaughlin N., Sharkey J., Pommey S., Denoyer D., Dwyer K.M., Smyth M.J. (2010). Anti-CD73 antibody therapy inhibits breast tumor growth and metastasis. Proc. Natl. Acad. Sci. USA.

[17] Antonioli L., Blandizzi C., Malavasi F., Ferrari D., Hasko G. (2016). Anti-CD73 immunotherapy: A viable way to reprogram the tumor microenvironment. Oncoimmunology.

[18] Leone R.D., Emens L.A. (2018). Targeting adenosine for cancer immunotherapy. J. Immunother. Cancer.

[19] Wang L., Fan J., Thompson L.F., Zhang Y., Shin T., Curiel T.J., Zhang B. (2011). CD73 has distinct roles in nonhematopoietic and hematopoietic cells to promote tumor growth in mice. J. Clin. Investig..

[20] Schürch C.M., Bhate S.S., Barlow G.L., Phillips D.J., Noti L., Zlobec I., Chu P., Black S., Demeter J., McIlwain D.R. (2019). Coordinated cellular neighborhoods orchestrate antitumoral immunity at the colorectal cancer invasive front. bioRxiv.

[21] Ehlerding E.B., England C.G., McNeel D.G., Cai W. (2016). Molecular Imaging of Immunotherapy Targets in Cancer. J. Nucl. Med.

[22] Frey E.C., Humm J.L., Ljungberg M. (2012). Accuracy and precision of radioactivity quantification in nuclear medicine images. Semin. Nucl. Med..

[23] Akahori Y., Kurosawa G., Sumitomo M., Morita M., Muramatsu C., Eguchi K., Tanaka M., Suzuki K., Sugiura M., Iba Y. (2009). Isolation of antigen/antibody complexes through organic solvent (ICOS) method. Biochem. Biophys. Res. Commun..

[24] Kurosawa G., Akahori Y., Morita M., Sumitomo M., Sato N., Muramatsu C., Eguchi K., Matsuda K., Takasaki A., Tanaka M. (2008). Comprehensive screening for antigens overexpressed on carcinomas via isolation of human mAbs that may be therapeutic. Proc. Natl. Acad. Sci. USA.

[25] Haun R.S., Quick C.M., Siegel E.R., Raju I., Mackintosh S.G., Tackett A.J. (2015). Bioorthogonal labeling cell-surface proteins expressed in pancreatic cancer cells to identify potential diagnostic/therapeutic biomarkers. Cancer Biol. Ther..

[26] Kurosawa G., Sumitomo M., Ukai Y., Subere J., Muramatsu C., Eguchi K., Tanaka-Hashiba M., Sugiura M., Ando M., Sato N. (2011). Selection and analysis of anti-cancer antibodies for cancer therapy obtained from antibody phage library. Cancer Sci..

[27] Hay C.M., Sult E., Huang Q., Mulgrew K., Fuhrmann S.R., McGlinchey K.A., Hammond S.A., Rothstein R., Rios-Doria J., Poon E. (2016). Targeting CD73 in the tumor microenvironment with MEDI9447. Oncoimmunology.

[28] Antonioli L., Yegutkin G.G., Pacher P., Blandizzi C., Hasko G. (2016). Anti-CD73 in cancer immunotherapy: Awakening new opportunities. Trends Cancer.

[29] Wiseman G.A., White C.A., Stabin M., Dunn W.L., Erwin W., Dahlbom M., Raubitschek A., Karvelis K., Schultheiss T., Witzig T.E. (2000). Phase I/II 90Y-Zevalin (yttrium-90 ibritumomab tiuxetan, IDEC-Y2B8) radioimmunotherapy dosimetry results in relapsed or refractory non-Hodgkin’s lymphoma. Eur. J. Nucl. Med..

[30] Fisher D.R., Shen S., Meredith R.F. (2009). MIRD dose estimate report No. 20: Radiation absorbed-dose estimates for 111In- and 90Y-ibritumomab tiuxetan. J. Nucl. Med..

[31] Olive K.P., Jacobetz M.A., Davidson C.J., Gopinathan A., McIntyre D., Honess D., Madhu B., Goldgraben M.A., Caldwell M.E., Allard D. (2009). Inhibition of Hedgehog signaling enhances delivery of chemotherapy in a mouse model of pancreatic cancer. Science.

[32] Provenzano P.P., Cuevas C., Chang A.E., Goel V.K., Von Hoff D.D., Hingorani S.R. (2012). Enzymatic targeting of the stroma ablates physical barriers to treatment of pancreatic ductal adenocarcinoma. Cancer Cell.

[33] Ryzhov S.V., Pickup M.W., Chytil A., Gorska A.E., Zhang Q., Owens P., Feoktistov I., Moses H.L., Novitskiy S.V. (2014). Role of TGF-beta signaling in generation of CD39+CD73+ myeloid cells in tumors. J. Immunol..

[34] Messaoudi N., Cousineau I., Henault D., McNicoll Y., Vandenbroucke-Menu F., Dagenais M., Letourneau R., Plasse M., Roy A., Lapointe R. (2018). CD73 as a novel immune target and biomarker in pancreatic adenocarcinoma. HPB.

[35] Argentiero A., De Summa S., Di Fonte R., Iacobazzi R.M., Porcelli L., Da Via M., Brunetti O., Azzariti A., Silvestris N., Solimando A.G. (2019). Gene Expression Comparison between the Lymph Node-Positive and -Negative Reveals a Peculiar Immune Microenvironment Signature and a Theranostic Role for WNT Targeting in Pancreatic Ductal Adenocarcinoma: A Pilot Study. Cancers.

[36] Fukamachi K., Tanaka H., Hagiwara Y., Ohara H., Joh T., Iigo M., Alexander D.B., Xu J., Long N., Takigahira M. (2009). An animal model of preclinical diagnosis of pancreatic ductal adenocarcinomas. Biochem. Biophys. Res. Commun..

[37] Morino K., Katsumi H., Akahori Y., Iba Y., Shinohara M., Ukai Y., Kohara Y., Kurosawa Y. (2001). Antibody fusions with fluorescent proteins: A versatile reagent for profiling protein expression. J. Immunol. Methods.

[38] Sogawa C., Tsuji A.B., Sudo H., Sugyo A., Yoshida C., Odaka K., Uehara T., Arano Y., Koizumi M., Saga T. (2010). C-kit-targeted imaging of gastrointestinal stromal tumor using radiolabeled anti-c-kit monoclonal antibody in a mouse tumor model. Nucl. Med. Biol..

[39] Yoshida C., Tsuji A.B., Sudo H., Sugyo A., Kikuchi T., Koizumi M., Arano Y., Saga T. (2013). Therapeutic efficacy of c-kit-targeted radioimmunotherapy using 90Y-labeled anti-c-kit antibodies in a mouse model of small cell lung cancer. PloS ONE.

[40] Snyder W.S., Nasset E.S., Cook M.J. (1975). Report of the Task Group on Reference Man: A Report Prepared by a Task Group of Committee 2 of the International Commission on Radiological Protection.

